# Study of Knowledge, Attitude and Practices Regarding Biomedical Waste among Paramedical Workers

**DOI:** 10.4103/0970-0218.66871

**Published:** 2010-04

**Authors:** Mohd Shafee, NB Kasturwar, N Nirupama

**Affiliations:** Dept. of Community Medicine, Chalmeda Anand Rao Institute of Medical Sciences, Bommakal, Karimnagar-505 001, Andhra Pradesh, India. E-mail: mohdshafee2008@gmail.com

Sir,

“Biomedical waste (management and handling) rules 1998” apply to all those who generate, collect, receive, store, transport, treat, dispose or handle biomedical waste and also to every institution that generates biomedical waste. This study was done to assess the knowledge, attitude and practices of hospital staff regarding biomedical waste management. Permission from head of the institution and clearance from Ethics Committee were obtained.

Karimnagar town (Andhra Pradesh) has a population of 5.2 lacs. It has 267 private nursing homes and clinics offering health services to the community. Out of 267 private nursing homes and clinics, 47 were selected by systematic random sampling. A total of 500 study subjects were selected from these hospitals and informed consent was obtained from them. The data were collected by one to one interview using pre-tested pre-designed proforma. The owner of three hospitals denied permission and five study subjects rejected to participate. This hospital-based cross-sectional study was performed from March to September 2009 in Karimnagar town. Statistical analysis was done using Chi-Square Test and percentage.

Out of 500 study subjects, 201 (40.2%) were males and 299 (59.8%) were females; 237 (47.4%) were nurses, 132 (26.4%) were lab technicians and 131 (26.2%) were housekeeping staff. Totally 266 (53.2%) study subjects knew about BMW correctly, of which 138 (51.8%) were nurses, 114 (42.85%) were technicians and 14 (5.26%) were housekeepers. Only 8 (1.6%) study subjects knew about categories of BMW of which 5 (62.5%) were technicians. Total 353 (70.6%) study subjects were having idea about segregation of BMW. Only 72 (14.4%) subjects had knowledge about various methods of disposal of BMW. Majority of the study subjects i.e. 479 (95.8%) had knowledge about various health problems caused by BMW, of which 234 (48.8%) were nurses.

The attitude of the study subjects toward separation of infectious and non-infectious waste, proper disposal and implementation of rules was positive i.e. 496 (99.2%), 494 (98.8%) and 492 (98.4%), respectively. Only 278 (55.6) study subjects committed that they will cooperate in BMW management. The nurses had a better attitude toward separation of wastes 236 (99.5%), proper disposal 234 (98.7%), implementation of rules 233 (98.3%) and cooperation in programs 149 (62.8%). The attitude of technicians and housekeeping staff was found to be almost similar.

Of the 482 (96.4%) study subjects who minimized waste, 227 (47%) were nurses, 129 (26.76%) were technicians and 126 (26.14%) were housekeepers. Totally 335 (67%) study subjects segregated BMW, of which majority were nurses, 169 (50.44%). Of the 297 (59.4%) subjects who collected waste into colour coded bags, 150 (50.5%) were nurses. Segregation and separation of plastic waste was done better by the nurses i.e. 169 (50.4%) of 335 (67%) and 56 (11.2%) of 95 (58.9%), respectively. None of the subjects disinfected the waste before disposal. Totally 490 (98%) subjects were sending BMW to private agency for disposal and treatment.

In this study, nurses had a statistically significantly better knowledge than the technical and housekeeping staff (*P*<0.001, *x^2^*=30.9). The study in a tertiary hospital showed that 85% nurses, 14% housekeeping and 12% technical staff had knowledge about BMW.(1) In Gujarat, it was found that doctors were aware of risk of health hazards, whereas auxiliary staff (ward boys, aayabens, sweepers) had very poor knowledge about it.(2) It was also found that the nurses had significantly positive attitude when compared to the technicians and the housekeeping staff (*x^2^* =64, *P*<0.02, df =1). In one of the study, it was found that 98% of the nurses and 79% of the shousekeeping staff had a positive attitude while only 59% of the technical staff had a positive attitude.(2)

Regarding BMW practices, it was found that the nurses practiced BMW management better than the technical and housekeeping staff and a significant difference was found (*x^2^*=9.48, *P*<0.01, df=1). Only 95 (19%) of the subjects collected plastic waste separately of which 56 (59.8%) were nurses. In a tertiary hospital, it was found that 100% nurses, 70% of the housekeeping staff and only 47% of the technical staff practiced BMW management.(1) At Jhansi it was found that the process of segregation, collection, transport, storage and final disposal of infectious waste was done in compliance with the standard procedures. It was also found that the non-infectious waste was collected separately in different containers and treated as general waste.(3) In Chandigarh, the medical establishments in the rural area and smaller ones in the urban area dispose off their biomedical waste along with municipal solid waste and no waste management system exists.(4) In one of the district in Gujarat, there was no effective waste segregation, collection, transportation and disposal system at any hospital.(1) In Karachi, it was observed that 25% hospitals were segregating sharps, pathological waste, chemical, infectious, pharmaceutical and pressurized containers at source.(5)

The staff lacked the required knowledge about BMW management. A positive attitude was found to improve the current situation in BMW management. The nurses were having better knowledge and attitude, and also practiced BMW management better than the housekeeping and technical staff. Regular training of nursing, technical and housekeeping staff should be done and system of monitoring should be evolved. Nursing staff who are correctly practicing BMW management should be involved as role models for others.
